# The C-terminal low-complexity domain involved in liquid–liquid phase separation is required for BRD4 function *in vivo*

**DOI:** 10.1093/jmcb/mjz037

**Published:** 2019-05-08

**Authors:** Chenlu Wang, Erhao Zhang, Fan Wu, Yufeng Sun, Yingcheng Wu, Baorui Tao, Yue Ming, Yuanpei Xu, Renfang Mao, Yihui Fan

**Affiliations:** 1 Department of Immunology, School of Medicine, Nantong University, Nantong 226001, China; 2 Laboratory of Medical Science, School of Medicine, Nantong University, Nantong 226001, China; 3 Department of Pathophysiology, School of Medicine, Nantong University, Nantong 226001, China

**Dear Editor**,

Recently, liquid–liquid phase separation (LLPS) attracts great interest for its ability to achieve spatial separation and effective organization of macromolecules (Gomes and Shorter, 2018). It is believed to be the driving force to form membraneless organelles and thus plays fundamental roles in a large number of biological processes (Shin and Brangwynne, 2017). A list of proteins have been identified that could undergo LLPS *in vitro* and *in vivo* (Du and Chen, 2018). However, the physiological role of LLPS in animals remains largely unknown.

BRD4 is a pivotal epigenetic reader that recognizes acetylated lysine residues. It belongs to the bromodomain (BD) and extra-terminal (ET) domain family (BET) that consists of four members including BRD2, BRD3, BRD4, and BRDT (Wu and Chiang, 2007; Andrieu et al., 2018). Members of the BET family contain two tandem BDs (BDI and BDII) and an ET domain (Supplementary Figure S1A). Interestingly, unlike the other three members, BRD4 has a unique C-terminal low-complexity domain (LCD) (Supplementary Figure S1A). BRD4-LCD not only forms phase-separated droplets *in vitro* but also forms phase-separated droplets at super-enhancer regions in living cells (Sabari et al., 2018). However, the physiological function of BRD4-LCD has not yet been investigated *in vivo*.

Normally, BRD4 encodes two isoforms via alternative exon usage. The long isoform (BRD4-L) contains an extended C-terminal LCD and a P-TEFb-interacting domain (PID). The short isoform (BRD4-S) shares almost the same amino acid sequence with BRD4-L but lacks C-terminal LCD and PID (Chiang, 2014; Supplementary Figure S1A and B). We established a mouse model with a specific deletion of the C-terminal LCD but with intact BRD4 N-terminus (BRD4^dLCD^; Figure 1A). BRD4^dLCD/+^ heterozygotes (Het) were obtained and genotyped (Figure 1B). To further examine the expression of BRD4-L and BRD4-S, we prepared mouse embryonic fibroblasts (MEFs) from day 13.5 embryos. Real-time reverse transcriptase-polymerase chain reaction (RT-PCR) clearly showed that the transcript from exons 12 to 20 but not exons 10 to 11 was significantly reduced in BRD4^dLCD/+^ MEFs (Figure 1C). Western blot analysis further demonstrated that BRD4-L was dramatically attenuated, while BRD4-S was slightly enhanced in BRD4^dLCD/+^ MEFs (Figure 1D). BRD4-S-specific antibody further confirmed that the expression of BRD4-S in BRD4^dLCD/+^ MEFs was enhanced (Figure 1E). It was reported that Aurka and Spc24 are regulated by BRD4-S, while Regnase-1 and PD-L1 are targets of BRD4-L (Alsarraj et al., 2011; Yang et al., 2018). We, therefore, examined the expression of these genes and found that deletion of the LCD did not affect the expression of BRD4-S-regulated genes but significantly downregulated the expression of BRD4-L-regulated genes (Figure 1F). Collectively, all of these results demonstrated that we have successfully established a mouse model that expresses BRD4 protein without the LCD.

Next, we crossed Brd4^dLCD/+^ with wild-type (WT) mice and genotyped 65 3-week-old mice. Only 12 of the heterozygous mice were obtained while the expected number should be 33 (Figure 1G), which indicates that ~64% (21/33) of Brd4^dLCD/+^ mice died within 3 weeks. To obtain homozygous (Brd4^dLCD/dLCD^) mice, we crossed Brd4^dLCD/+^ with Brd4^dLCD/+^ mice. We genotyped 16 3-week-old mice and found 10 WT, 6 Het, and no homozygotes (Supplementary Figure S1C). This suggests that 70% Het died and none of null-knockout mice survived. The surviving BRD4^dLCD^ Het were smaller than WT littermates. The average weight of 8-week-old WT and BRD4^dLCD^ heterozygous mice are 20.5 g and 17.1 g, respectively (Figure 1H). The Het also showed an abnormal shape of their heads with shorter and bent nasal bones (Figure 1I and J). The phenotypes observed in BRD4^dLCD^ mice are identical to the reported phenotypes in BRD4-knockout mice (Houzelstein et al., 2002). To further characterize the *in vivo* functions of the LCD particularly the critical role of LLPS in neurodegenerative diseases, the working memory of BRD4^dLCD/+^ and WT mice was assessed by using alternative electro-stimulus Y maze. The error times of BRD4^dLCD/+^ mice after 3-day training is significantly higher than that in WT mice (Figure 1K). We also measured the tail flicking withdrawal response to an infrared heat stimulus in BRD4^dLCD/+^ and WT mice. The latency of tail-flicking in BRD4^dLCD/+^ is significantly longer than that in WT mice (Figure 1L). Taken together, the above results revealed an essential role of the BRD4 C-terminal LCD *in vivo*.

**Figure 1:**
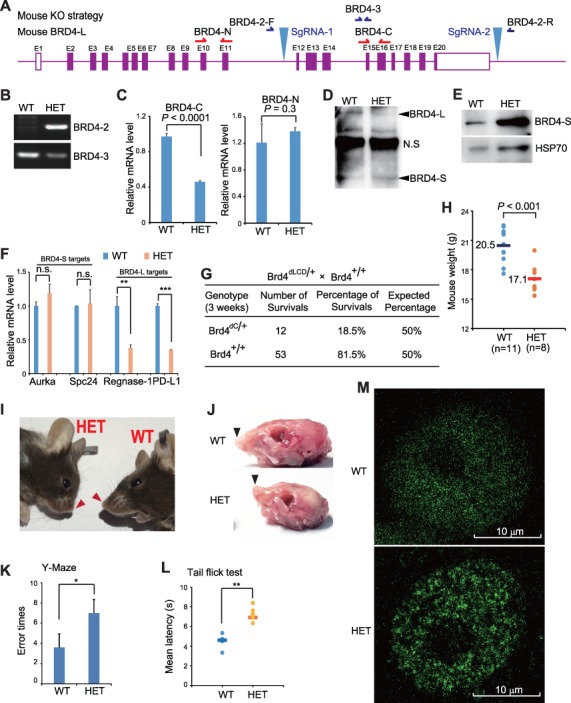
C-terminal LCD domain is required for BRD4 function *in vivo*. (**A**) Schematical representation of CRISPR/Cas9-mediated deletions of exons 12 to 20 of the BRD4 gene in mice. (**B**) Genotyping of BRD4^+/+^ (WT) and BRD4^dLCD/+^ (Het) by using BRD4-2 and BRD4-3 primers. (**C**) Real-time RT-PCR was used to detect mRNA of full-length or C-terminal LCD-deleted BRD4 mutants in WT and Het mice. (**D**) Western blot analysis was performed to examine the expression of BRD4-L and BRD4-S in WT and Het mice. (**E**) Western blot analysis was performed to examine the expression of BRD4-S in WT and Het mice. (**F**) Real-time RT-PCR was used to detect the mRNA of BRD4-L-regulated genes (Regnase-1 and PD-L1) and BRD4-S-regulated genes (Aurka and Spc24). (**G**) Genotype of 3-week progeny from crossing between BRD4^dLCD/+^ and BRD4^+/+^ mice. (**H**) The weight of 8-week-old BRD4^dLCD/+^ (*n* = 8) and BRD4^+/+^ (*n* = 11) mice. (**I**) Representative image of heads of BRD4^dC/+^ and BRD4^+/+^ mice. (**J**) Representative image of shorter and bent nasal bones of BRD4^dC/+^ mice. (**K**) The alternative electro-stimulus Y maze was used to measure the working memory of BRD4^dLCD/+^ and WT mice. (**L**) The latency of tail-flicking in response to an infrared heat stimulus in BRD4^dLCD/+^ and WT mice was measured. (**M**) Immunofluorescence using anti-BRD4 antibodies (ab128874) was performed to determine the distribution of BRD4 in the nucleus in BRD4^dC/+^ and BRD4^+/+^ MEFs. *P<0.05; **P<0.01; ***P<0.001. n.s., no significance.

To further validate our finding, we specifically deleted the C-terminal LCD in human embryonic kidney 293T cells. The result of genotyping indicated deletions of exons 12 to 17 (Supplementary Figure S2A and B). In addition, western blot analysis showed that the expression of BRD4-L but not BRD4-S was significantly decreased in the knockout cells relative to control cells (Supplementary Figure S2C). The proliferation and colony formation of sgBRD4-dE12-E17 cells were dramatically reduced compared to that of control cells (Supplementary Figure S2D and E). These results are in line with the previously reported BRD4 function in human cells.

The C-terminal LCD of BRD4 undergoes phase separation *in vitro* and BRD4 forms puncta as membraneless organelles. Unexpectedly, deletion of the C-terminal LCD in mice and in human cells results in enlarged BRD4 puncta (Figure 1M; Supplementary Figure S2F). One possible explanation is that the LCD drives LLPS and prevents BRD4-S accumulation at specific locations. The ratio of BRD4-L and BRD4-S in BRD4 puncta is important for maintaining puncta in a proper size to avoid a viscous gel phase. Another possibility is that the observed BRD4 puncta is not generated through LLPS. The molecular mechanism regarding the enlarged BRD4 puncta after deletion of the LCD needs to be further addressed. Taken together, our data provide compelling evidence to demonstrate that the C-terminal LCD is critical for BRD4 functions both in mice and in human cells.

*[Supplementary material is available at *Journal of Molecular Cell Biology* online. We thank Prof. Cheng-Ming Chiang from UT Southwestern Medical Center for editing the manuscript. This work was supported by the National Natural Science Foundation of China (81641164, 81600386, 31770935, and 81873531), the Distinguished Professorship Program of Jiangsu Province (to R.M.), the Natural Science Foundation of Jiangsu Province (BK20181458), and the Postgraduate Research & Practice Innovation Program of Jiangsu Province (KYCX18_2403).]*

## Supplementary Material

JMCB-2018-0529_R2_Supplementary_Material_mjz037
