# Supplementary material for: The C-terminal low-complexity domain involved in liquid–liquid phase separation is required for BRD4 function in vivo
Source: J Mol Cell Biol. 2019 May 8;11(9):807–9. doi: 10.1093/jmcb/mjz037 (PMC6821289; doi:10.1093/jmcb/mjz037)
Supplement: JMCB-2018-0529_R2_Supplementary_Material_mjz037 [file jmcb-2018-0529_r2_supplementary_material_mjz037.pdf]

The C-terminal low-complexity domain involved in liquid-liquid phase separation is required for BRD4 function in vivo

Chenlu Wang [1], Erhao Zhang [2], Fan Wu [2, 3], Yufeng Sun [2], Yingcheng Wu [2], Baorui Tao [2, 3], Yue Ming [1], Yuanpei Xu [1], Renfang Mao [2, 3, *], Yihui Fan [1, 2, *]

[1] Department of Immunology, School of Medicine, Nantong University, Jiangsu, 226001, China.
[2] Laboratory of Medical Science, School of Medicine, Nantong University, Jiangsu, 226001, China.
[3] Department of Pathophysiology, School of Medicine, Nantong University, Jiangsu, 226001, China.

*** Address correspondence to:**

Renfang Mao, M.D., Ph.D.
Department of pathophysiology, School of Medicine, Nantong University,
19 Qixiu Road, Nantong, People's Republic of China, 226001,
Email: maorenfang@ntu.edu.cn

Yihui Fan, M.D., Ph.D.,
Laboratory of Medical Science, School of Medicine, Nantong University,
19 Qixiu Road, Nantong, China, 226001,
Email: fanyihui@ntu.edu.cn

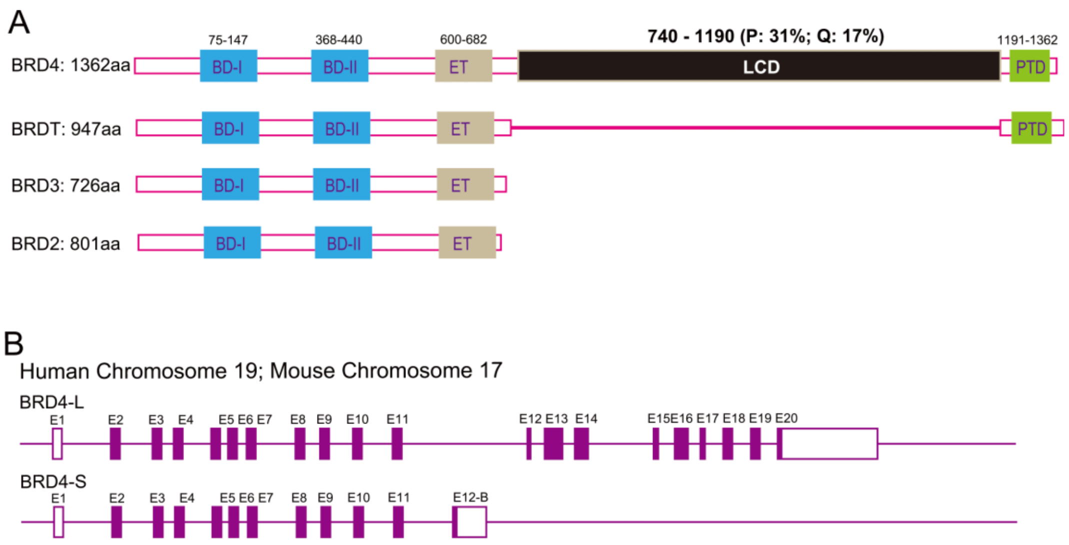

C

$Brd4^{dLCD}/+$ x $Brd4^{dLCD}/+$

Genotype (3 weeks)	Number of Survivals	Percentage of Survivals	Expected Percentage
$Brd4^{dLCD/dLCD}$	0	0%	25%
$Brd4^{dLCD}/+$	6	37.5%	50%
$Brd4^{+/+}$	10	62.5%	25%

Figure S1

(A) Schematical representation of domain organization of BET family members including BRD4, BRDT, BRD3 and BRD2. (B) Schematical representation of the exon organization of different BRD4 isoforms (BRD4L and BRD4S). (C) Genotype of progeny from crossing between $BRD4^{dC/+}$ and $BRD4^{dC/+}$ mice at three weeks old.

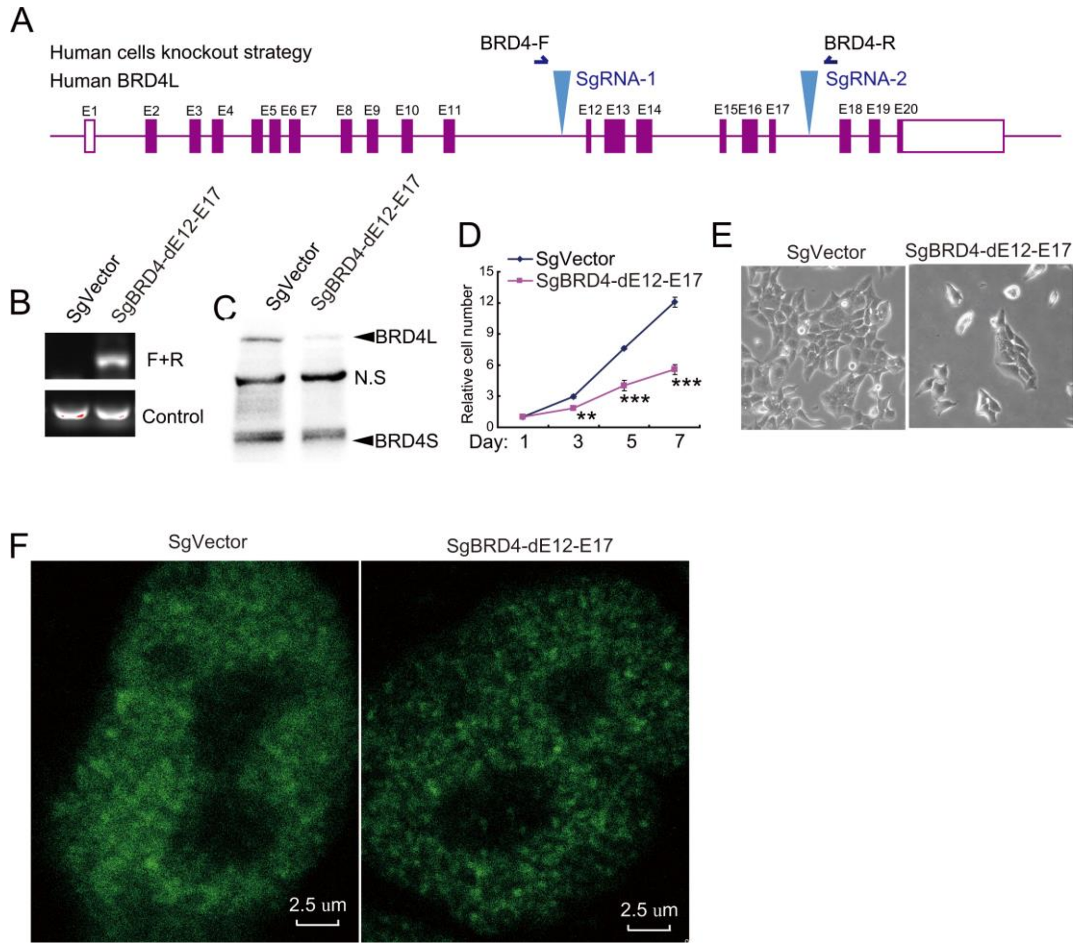

Figure S2

(A) Schematical representation of the design of CRISPR/Cas9 mediated deletion of the exons 12 to 17 of BRD4 gene in human cells. The location of genotyping primers BRD4-F and BRD4-R were also indicated. (B) Genotyping of BRD4 exon 12 to 17 deleted cells (SgBRD4-dE12-E17) using BRD4-F and BRD4-R primers. (C) Western blot analysis was performed to examine the expression of BRD4 long isoform and short isoform in sgVec and sgBRD4-dE12-E17 cells. (D) Proliferation curve of sgVec and sgBRD4-dE12-E17 cells. (E) Colony formation of sgVec and sgBRD4-dE12-E17 cells. (F) Immunofluorescence using anti-BRD4 antibodies (ab128874) was performed to determine the distribution of BRD4 in nucleus from sgVec and sgBRD4-dE12-E17 293T cells.
